# Buying electricity resilience: using backup generator sales in the United States to understand the role of the private market in resilience

**DOI:** 10.1186/s43065-023-00078-5

**Published:** 2023-05-06

**Authors:** Daniel Thompson, Gianluca Pescaroli

**Affiliations:** Institute for Risk and Disaster Reduction, University College London, London, UK

**Keywords:** Electricity resilience, Backup generators, Private demand, United States, Household resilience

## Abstract

Disruptions to key lifelines, especially electrical power, can cause outsized impacts on human functioning. The state of the art on developed countries has focused on enhancing resilience to electrical grid infrastructure but has neglected to track changes regarding how the private market has developed electricity continuity measures over time. Backup generators are among the most accessible tools to maintain electricity continuity in case of power failure, but their role as a buffer remains understudied outside the technical domain, along with the humanitarian and emergency response sectors.

This paper analyzes generator sales across the U.S. to understand some underlying trends that may have influenced changes in consumer preference for electricity resilience. Reports from major backup generator sellers and import data of backup generators reveal an increase in backup generators across the U.S. and find that private demand for energy resilience is likely increasing due to consumers’ perceived risk and rising levels of intolerance to power disruptions. The discussion finds that an increase in private demand and use of backup generators may be impacting electricity resilience at a communal and societal level, which seems to be underexamined by studies focusing on private generator usage in the U.S..

## Introduction

The importance of maintaining a continuous electricity supply has received increasing attention from researchers over the past two decades. Strengthening electrical systems can minimize the frequency and duration of disruptions; however, this approach seems to have overlooked the importance of individual behaviors to buffer against these events. This paper explores changes in private demand for backup generators across the United States as a proxy to measure changes in private behavior toward electricity resilience in developed countries.

The state of the art on electricity resilience builds from the well-established premise that system failures, including electricity disruptions, cause outsized impacts on human functioning. This paper defines any deviation from a continuous supply of electrical energy as a disruption. A disruption is a power outage, failure, or blackout that results in a partial or complete loss of power supply to an end user. Causes of disruptions can include natural hazards, human error (unintentional), and deliberate human sabotage [1].

Recent theories of cascading risk have demonstrated how a disruption could produce an “escalation point” that would elevate the intensity of a crisis [2]. Further studies have noted that cascading risk in electrical infrastructure systems may accelerate over time due to increasing interconnectivity of these systems and consumers’ growing dependence on them for basic functions [3]. Resilience approaches are well suited to address dynamic threats and the interconnected nature of electricity systems by buffering, recovering, and transforming from adverse events [4]. To meet these challenges, resilience studies have contributed to the development of new approaches, tools, and strategies. The recent application of stress testing approaches from other sectors to infrastructure, for example, could enhance understandings of system components and prioritize resilient actions [5].

Electrical resilience is a subset of resilience research that focuses on strengthening electrical systems. Many of these studies focus on electrical grids exclusively [6]. Fewer studies emphasize the role of backup generators and other buffering strategies in maintaining a continuous supply of power. Backup generators can be classified by their technology and energy input. Most private backup generators can be classified as photovoltaic, internal combustion (gas and diesel), fuel cells, and electricity storage (various battery types), or a combination of these generators [7]. These four classifications of backup generators are the focus of this study.

Resilience studies of backup generators can be divided into two general categories. The first category takes a system-wide approach by focusing on how backup generators can improve or maintain electricity supply to electrical grids and critical infrastructure. Energy distribution studies and emergency management studies have contributed significantly to this category of study. Technical and theoretical research on energy distribution has enhanced understandings of how backup generators can augment critical infrastructure systems, including the transition to renewable energy sources [8]. There is also robust technical research on microgrids that explores how to optimize distributed generator systems, including backup generators, to enhance the resilience of electrical systems [9]. A subset of optimization studies have investigated the business case for microgrids as a resilient investment, with some studies finding that revenues generated by microgrids could not cover the cost of investment [10]. Emergency management and humanitarian studies highlight the importance of generators in emergency planning and in maintaining electrical power for critical facilities to prevent cascading failures [11, 12]. There is minimal overlap with this category of study and the second category.

The second category of study examines how private individuals use backup generators to maintain a continuous supply of power during emergency events. The second category lacks the systemwide approach that characterizes first category. Although this category of study has received comparatively less attention than the first category, research on private demand has increased in the last several years. Several case studies focusing on the immediate impacts of disruptions have highlighted equity concerns, such as how some households struggle to survive, while other wealthy households buffer their exposure to the disaster by vacating the outage area [3]. Preparedness research seems to have paid more attention to the use of backup generators as redundancy measures [13]. Threshold analysis of household tolerance to power outages also has concluded that backup generators could improve household conditions during power outages [14, 15] On the commercial side, there exists a well-established body of literature on backup generator use among businesses, particularly focusing on developing countries [16]. This may be due to the outsized role that generators and generator inputs play in buffering electrical outages in emerging economies, as a recent study estimated that 137 low- and middle-income countries spend $64 billion (USD) annually on backup generators and inputs [17]. For some comparison, a report estimates that only 5% of the U.S. residential market owns a backup generator as of 2020 [18].

Despite the proliferation of recent studies that focus on private demand for backup generators in the second category, research that traces shifts in private demand over time has been comparatively overlooked, especially in the United States [19, 20]. One of the most notable studies on this topic found that Hurricane Rita prompted some commercial enterprises in southeastern Texas to purchase backup generators [21].

This paper explores changes in private demand for backup generators in the U.S., since the U.S. could provide insight into potential trends in countries that share comparable electrical grids and economic conditions. The U.S. electric grid is composed thousands of private, public, and private–public entities that control aspects of the generation, transmission, and distribution of electricity. A patchwork of agencies spanning from the local to the federal government regulate this highly interconnected grid. Clark-Ginsberg et. al have noted these similarities in Continental Europe [22]. The U.S. and Europe’s electric grids are also aging, which have contributed to failure events. Despite economic differences, the U.S. and Europe share similar consumer purchasing power indices and access to goods [23]. These characteristics may play a role in driving generator demand since consumers have the willingness (due increasing electrical failure) and the ability to purchase backup generators. Although early studies suggest similarities between the U.S. and established economies, more research is needed to understand these trends in similar countries [24].

This paper seeks to answer the question: *what would an increase in private demand for backup generators in the United States imply about private attitudes toward electrical resilience in the U.S.?* A mixed-method analysis of backup generator sales and import data of backup generators in the U.S. suggests that private demand has increased over the last several years, which could be attributed to consumers’ increased perceptions of risk and rising levels of intolerance to electricity disruptions. This paper seeks to add to the second area of backup generator study by highlighting the importance of long-term demand shifts for backup generators, which could impact the conclusions in some of these studies. This paper also seeks to bridge a theoretical gap between the two categories of study by arguing that increased private demand for generators could present an opportunity to strengthen the resilience of electrical systems.

The paper is organized into six subsequent sections. The proceeding section outlines the paper’s hypothesis and methods by providing a rationale for the data examined, along with some of its limitations. The following two sections highlight the results of the study, as the third section suggests that private demand for backup generators has increased in the last several years while the fourth section posits reasons for this shift. The fifth section discusses some potential societal and systemic consequences of increased private generator demand. The conclusion section recapitulates the study’s key messages and presents several areas for future research.

## Methods

Given a lack of consistently recorded data on private backup generator use or ownership in the United States, sales data and trade data on backup generators was selected as a proxy for demand. The study focused on commercial, residential, and industrial markets segments since these segments have minimal regulatory mandates to purchase backup generators, meaning that most purchases in these segments are voluntary. *The research approach was structured into two parts, part A and part B, to address the two-part research question: are generator sales increasing (A) and, if so, why are they increasing (B)?* This paper hypothesizes that demand for backup generators is increasing, which is being driven, in part, by consumers’ perceptions of risk and increased risk intolerance.

Part A uses an embedded mixed-method approach of backup generator sales data from major generator sellers in the U.S. and quantitative U.S. import data of backup generators as proxies for changes in demand. In an embedded mixed-method approach, either qualitative or quantitative data is secondary the other [25]. Studies have shown how an embedded mixed-method approach can surmount data limitations, which in this case is a general lack of publicly available data, especially quantitative data, on backup generators in the United States [26]. This study uses quantitative sales and important data to support an analysis of qualitative reporting from backup generator companies. Qualitative data was collected from an analysis of companies written reports over the past several years.

Search engine and database queries, correspondence with data specialists, and other studies identified reports from backup generator companies as a way to track historical generator sales. An examination of listings from consumer reports, private reports, and internet queries revealed nine generator companies as among the largest players in the U.S. market, which is shown in Table 1. Each of these companies was analyzed to determine if their reports contained qualitative or quantitative data on backup generator sales. Four companies, Briggs and Stratton, Cummins, and Generac, and Honda included qualitative data on backup generator sales, with only Cummins and Generac reporting qualitative data as well. Variability across qualitative and quantitative reports makes it difficult to determine the collective market share of the four companies. Nonetheless, a relatively conservative analysis finds that the four companies would constitute a majority (at least 60%) of the market [27]. For all quantitative data with an associated monetary value, price variability was reduced by converting into 2021 U.S. dollars [28]. Data from this query were used as the basis of research in Part A and Part B.

**Table 1:** U.S. Backup Generator Companies Reviewed

Company	Publicly Available Report	Qualitative Sales Data Reported	Quantitative Sales Data Reported
**Briggs and Stratton**^**a**^	Yes	Yes	No
Caterpillar	Yes	No	No
Champion	No	-	-
**Cummins, Inc.**^**a**^	Yes	Yes	Yes
**Generac**^**a**^	Yes	Yes	Yes
**Honda**^**a**^	Yes	Yes	No
Honeywell	Yes	No	No
John Deere	Yes	No	No
Kohler (Power)	No	-	-

^a^Selected for analysis based on data availability

Data from generator companies were augmented with trade data on U.S. imports of generators from 1991 – 2021, using the UN Comtrade database (Comtrade data). All AC and DC generators were used in the analysis, except for the category of the largest generators for both categories (DC > Kilowatts and AC > 750 Volt Amps), since most generators purchased by the private sector tend to not exceed these limits [18, 29–34]. Indeed, the category of the smallest generators in terms of kilowatts and amps reported the largest trade values. Comtrade ID numbers for these generator types include 850,131, 850,132, 850,133, 850,161, 850,162, and 850,163. Trade values in USD were used instead of numbers of generators, since unit numbers and other units of measurement were inconsistently reported across years.

Like Part A, Part B also uses a mixed method approach with quantitative data supporting qualitative reports from backup generator companies. In addition to quantitative data limitations, this approach was selected mainly because much of the qualitative data collected provided clear reasons for the change in backup generator demand. Statements from the 4 generator companies identified in Part A were used as the basis for the qualitative analysis.

For the quantitative analysis, Comtrade data developed in Part A was analyzed to determine its potential relationship to electrical reliability measures and other macroeconomic indicators in the United States, which supported the qualitative rationale provided by backup generator companies. Comtrade data were selected over sales data from generator companies since Comtrade data were more comprehensive over more years.

The Energy Information Administration (EIA) has collected and collated data on standard reliability measures in the United States since 2013, which were used as the electrical reliability metrics for this analysis. Three reliability measures across two scenarios were used for a total of 6 factors examined. EIA’s reliability measures adhere to IEEE-1366 standards (Institute of Electrical and Electronics Engineers-1336). The System Average Interruption Frequency Index (SAIFI) represents the average number of disruptions that customers experienced for the year, which is measured by aggregating the number of customers affect by a disruption out of total customers in a system. The System Average Interruption Duration Index (SAIDI) represents the average number of minutes that affected customers experienced a disruption for the year. These two measures can be derived into a final measure, Customer Average Interruption Duration Index (CAIDI), which represents the average number of minutes to restore power to customers that year. The two scenarios examined included data all disruptions (including major event days; MED) and data without major event days (normal; NOR) [35] (Table 2).

**Table 2:** Electricity Reliability Metrics (IEEE 1366) [36]

SAIFI = $$\frac{{\sum }_{i=1}^{n}{N}_{i}}{{N}_{T}}$$
SAIDI = $$\frac{{\sum }_{i=1}^{n}{{r}_{i}N}_{i}}{{N}_{T}}$$
CAIDI = $${\sum }_{i=1}^{n}\frac{{{r}_{i}N}_{i}}{{N}_{i}}$$

n: Number of events in a reporting period

N_i_: Number of customers without power for each disruption event

N_T_: Total number of customers served

r_i_: Restoration time for each disruption event

Both scenarios for each reliability measure (MED and NOR) were adjusted to reflect historical change over time and compared with changes in Comtrade data over time to highlight correlations (with 1991 = 0). A single-variable linear regression analysis was conducted between trade value (dependent variable) and each reliability measure (independent variable) to determine correlation. A single-variable regression was selected to isolate the impact of each factor on Comrade data since all three reliability measures are interdependent.

Comtrade data also was analyzed against macroeconomic indicators from the U.S. Federal Reserve data (FRED) and the EIA to determine their impact on generator trade values. U.S. Federal Reserve data (FRED) indicators selected include real medium household income (ID: MEHOINUSA672N), real final household consumption (ID: NCPHIRSAXDCUSQ), and the period of U.S. Recessions (ID: JHDUSRGDPBR) [37–40]. This analysis also selected EIA data on the total number of residential and commercial electricity customers in the U.S. to determine the impact of market growth on generator demand. Like reliability metrics, macroeconomic indicators were adjusted for change over time (with 1991 = 0) to smooth disparities between absolute data values and plotted with Comtrade data to highlight correlations. A single-variable regression was selected to isolate the impact of each factor on Comtrade data. Multiple-variable regression linear analyses of these macroeconomic indicators were conducted and were found to be insignificant (F Statistic < *p* value for all indicators). For this and other reasons, qualitative analysis was used to support available quantitative data.

This paper relies on several assumptions and has several limitations that should caution an interpretation of the results. Data profiled includes the entire U.S. since sales and import data did not segment sales by state or other geographic division. In addition to geographic limitations, the paper did not convert sales numbers (in $USD) into an estimated number of generators purchased due to the expansive range in generator pricing.

None of the generator companies reported major changes in product offerings that would have resulted in significant changes in sales revenue, which is also assumed for trade data. Some of this data includes backup generators for government applications, which is outside the scope of this study. An analysis of these companies’ offerings indicates that government sales were a minimal portion of their sales revenue. This assumption also applies to trade data. Specific backup generator companies presented limitations. Most prominently, Cummins only provided global quantitative data, not U.S. specific data. This paper cross-checked Cummins’s data with qualitative statements from Cummins’s reports U.S. sales trends, which suggest that global qualitative data generally match Cummins’s U.S. sales trends [41–47].

The lack of data and potential confounds in the data should caution an interpretation of these results. More research is needed on sales data, surveys, or other sources to determine the specific size and scope of generator demand and to isolate the specific impacts of potential causal factors, including risk perceptions and intolerance to outages. Despite these limitations, the methods selected can demonstrate simple trends (increasing, decreasing, static, etc.) of backup generator purchases and offers some reasons to explain these trends, which is the goal of this study.

## Results, part A: recent trends in backup generator demand

A qualitative and quantitative analysis of year-by-year sales from backup generator companies does not indicate a significant increase in backup generator sales over the last several years. Assuming that any portion of these sales are “new customers,” however, indicates that overall demand for backup generators has increased during this period. This paper aggregates available quantitative data into a model and runs simple regression analyses to show that assuming some number of new customers would result in an increase in overall backup generator demand over the past several years. Comtrade import data offer a much clearer increase in demand for generators over time.

Qualitative statements from company reports indicate that backup generator sales have not increased steadily over the last several years. Given the lack of quantitative sales data across the companies, qualitative reports about generator sales numbers were analyzed year-by-year and converted into Table 3. Red bars in the table denotes fewer sales compared to the previous year and green bars denotes more sales compared to the previous year. Briggs and Stratton, Cummins, and Honda share similar sales trends, which these reports attribute to changes in consumer demand. When framing Generac’s data against Briggs and Stratton and Honda’s qualitative reports, Generac’s sales may be more unusual, but too few companies were analyzed to indicate a conclusive result. These reports do not suggest significant vacillations in sales (more than 50%) year-by-year, which is supported by quantitative data from Generac and Cummins [18, 29–34, 41–61].

**Table 3:**
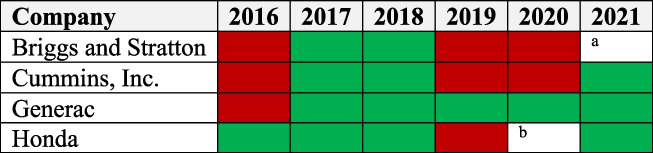
Change Value of Backup Generators Sold, Year over Year

^a^Briggs and Stratton delisted from the NYSE and did not provide another report on generator sales later than Q2 2020. 2020 data is from Q2

^b^Honda repeated information about 2019 generator sales in its 2020 report

**Fig. 1:**
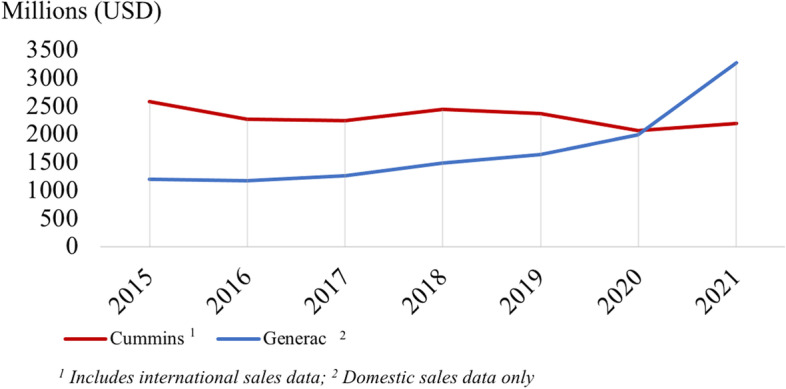
Yearly Historical Backup Generator Sales, 2015 – 2021

Year-by-year quantitative data in Fig. 1 supports the results from Table 3, as it indicates that annual sales have remained relatively constant over the recent 5-year period [41–47]. Cummins’s sales numbers have not increased or decreased dramatically year-by-year. Generac’s sales, by contrast, have climbed steadily over the last six years, followed by a rapid increase in 2021 [18, 29–34].

All companies except Generac did not report year-by-year sales increases, but these sales numbers still could imply strong demand for generators when considering that many purchasers would be new customers. “New customers” means any customer who does not purchase a generator to replace an existing generator unit, not only a customer who purchases their first generator. Lifespans for backup generators vary based on application and use, but most range between 10 and 30 years. Generator lifespans suggest that many generator purchasers could be first-time buyers.

**Fig. 2:**
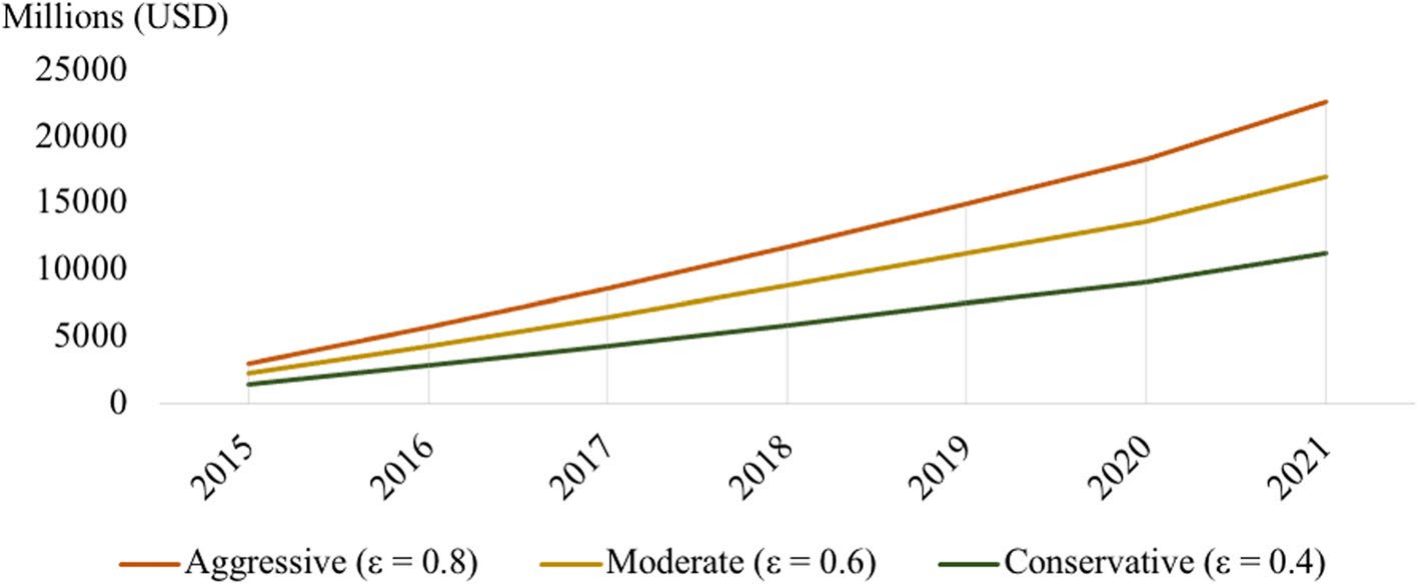
Aggregate Demand for Backup Generators in USD, 2015–2021

Although the number of first-time generator customers remains unknown, aggregating quantitative data from Generac and Cummins indicate that any number of first-time generator purchasers would constitute an increase in generators sales over this period. Sales data from Generac and Cummins (Fig. 1) were aggregated based on expected number of new customers (ε) as a portion of all yearly sales (x). Figure 2 displays three modeled estimates of generator demand based on 80% of new customers (aggressive; ε = 0.8), 60% new customers (moderate; ε = 0.6), and 40% new customers (conservative; ε = 0.4).

**Equation 1** Best-fit Regression for Model of Aggregate Demand (2015–2021)

**Table:** 

Linear best-fit analysis: f($\upvarepsilon$) = m$\upvarepsilon$x – b	m = 4022.5 b = m/4.56	$$\left\{\begin{array}{c}\\ \mathrm{x}>0\\\upvarepsilon =(\mathrm{0,1}]\end{array}\right\}$$	y = 4 $022.{5}\upvarepsilon$x – m/4.56	*R* ^*2*^ = 0.995
Power best-fit analysis: f($\upvarepsilon$) = α$\upvarepsilon$ × ^1.049^	α = 3644.25		y = 3644.25 $\upvarepsilon$× ^1.049^	*R* ^*2*^ = 0.996

The data also were modeled using linear and power best-fit regressions, since these two analyses best fit the data (see R2 analysis in Eq. 1). Simple linear and power models suggest that demand for generators is increasing steadily (note the positive values for m and α, along with β), which assumes that at least a small number of customers did not buy a generator as a replacement (ε > 0). In fact, both models indicate an increase in aggregate sales as long as some purchasers are new customers (expressed as ε > 0 → m > 0, α > 0). Determining the number of new customers would improve the accuracy of aggregate generator numbers; however, it does not alter the overall trend, which indicates an increase in total generator purchases over these years.

**Fig. 3:**
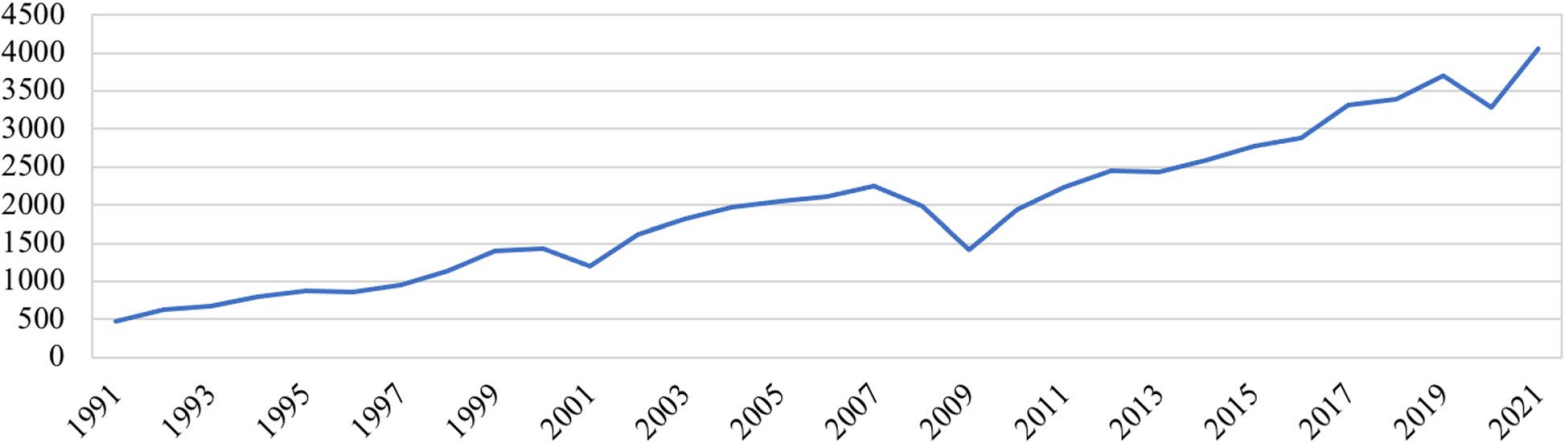
Import Value of Backup Generators in USD, 1991—2021

UN Comtrade data on backup generator imports also supports this conclusion, as the monetary value of generators has risen steadily, increasing nearly tenfold from 1991 to 2021 [62] (Fig. 3). The trend in the figure highlights an increase even before considering than many of these final customers for these products could be new customers. Given the uncertainty of determining a proportion of customers across a longer timeframe than several years, a new customer analysis was not conducted.

## Results, part B: reasons for increases in backup generator demand

Quantitative results on the reason for backup generator demand emphasize strong correlations between one electrical reliability indicator to the Comtrade dataset and almost all the macroeconomic indicators analyzed to the Comtrade dataset; however, results were insufficient to demonstrate causality. Results also indicate that purchases of backup generators have far exceeded macroeconomic growth. Qualitative indicators from primary and secondary sources suggest that consumers’ risk perception of disruptions and their rising intolerance to disruptions may be driving some demand for backup generator purchases. In this analysis, a growing contingent of consumers are manifesting suspicions that traditional electrical infrastructure cannot guarantee electrical power at tolerable level.

Risk perception, in this case, places primacy on how consumers assess their likelihood of experiencing an outage, which may or may not correlate with a specific frequency or duration of outages, at least as suggested in the macro-analysis of reliability data. The second concept, intolerance, builds from existing theories to argue that consumers’ intolerance to an electrical outage has increased because their dependence on electricity has increased. This increase in demand for backup generators can be considered as a manifestation of rising intolerance to power outages.

### Quantitative analysis – electricity disruptions

**Fig. 4:**
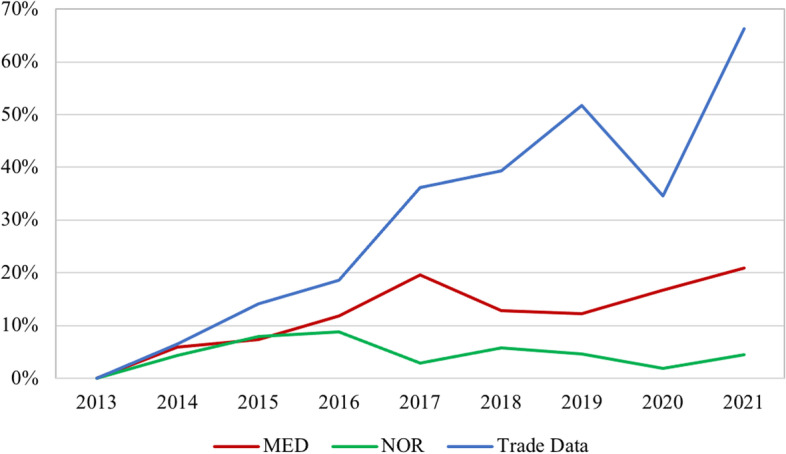
SAIFI Relationship to Import Value of Backup Generators (Percent Change), 2013—2021

**Fig. 5:**
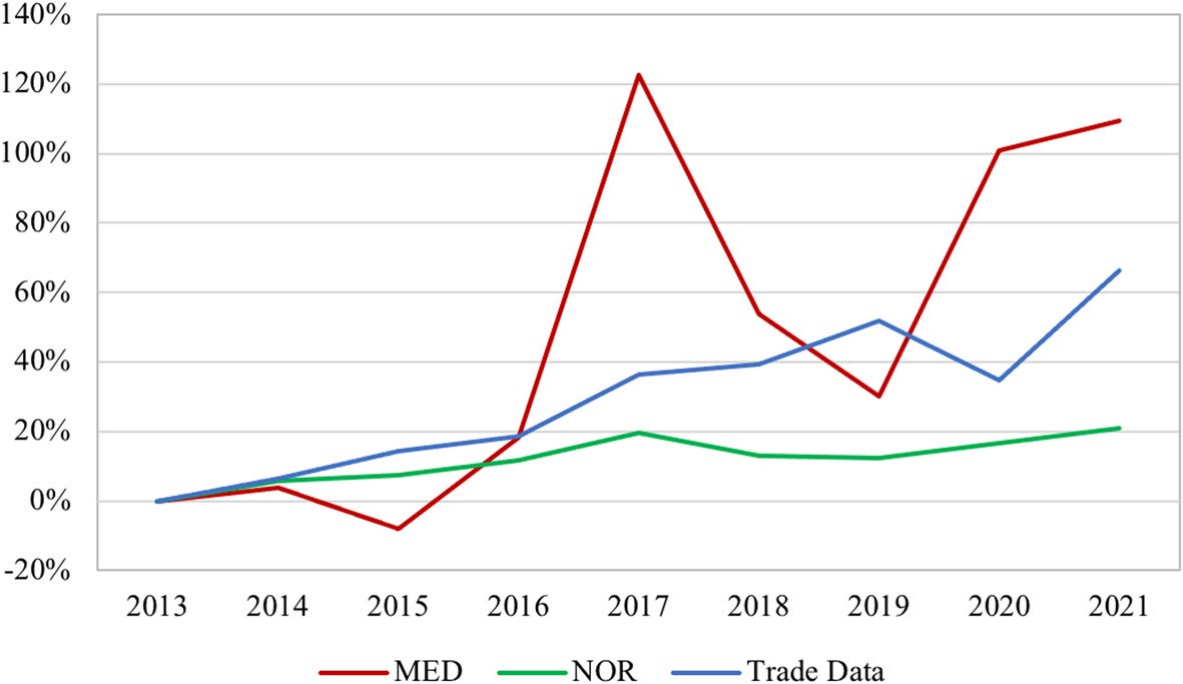
SAIDI Relationship to Import Value of Backup Generators (Percent Change), 2013—2021

**Fig. 6:**
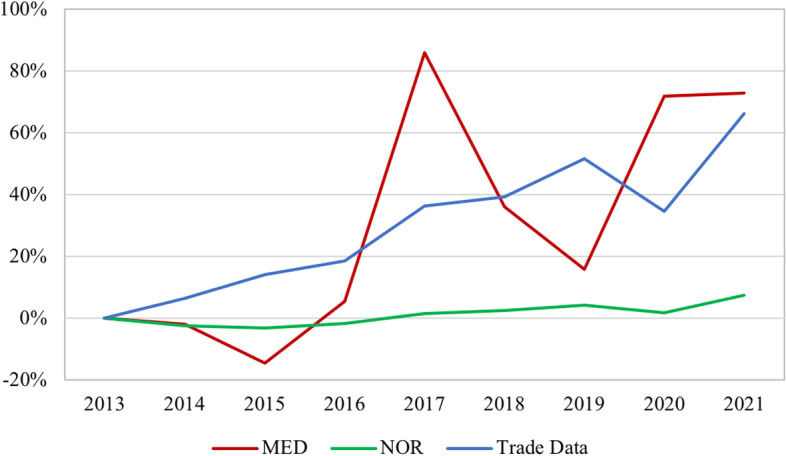
CAIDI Relationship to Import Value of Backup Generators (Percent Change), 2013—2021

Figures 4, 5 and 6 compare relative rates of change from Comtrade data (“trade data”) and SAIDI, SAIFI, and CAIDI from 2013 – 2020. Reliability measures are segmented into normal disruptions and major event days (MED) and normal disruptions only (NOR). The analysis reveals that almost none of the reliability measures relate to Comtrade data. Only CAIDI NOR was strongly correlated with Comtrade data, as indicated in the regression analysis in Table 4. The coefficient and standard errors for all variables were large, which can be explained by the disparity in the absolute numerical values between reliability measures (0 < *n* < 1000) and Comtrade values (1,000,000 < n). An analysis of these data did not indicate a causal relationship between power outages and increased purchases of backup generators.

**Table 4:** Regression Analyses of Import Value of Backup Generators to Electrical Reliability Meaures

Reliability Measure	Pearson Correlation	R^2^	Coefficient	Standard Error (Coefficient)	*p*-value
SAIFI (MED)	0.657	0.431	-2853.087	3075.735	0.396
SAIDI (MED)	0.550	0.302	2501.581	450.268	0.003
CAIDI (MED)	0.547	0.300	2414.666	510.020	0.005
SAIFI (NOR)	0.428	0.184	10,099.137	6570.513	0.185
SAIDI (NOR)	0.682	0.466	-7488.821	5090.521	0.201
CAIDI (NOR)	0.972	0.945	-10,423.596	1457.069	0.001

To account for potential market friction, Comtrade data was staggered by one and two years and compared to the 2013—2021 electricity reliability data set. In the one-year staggered model, for instance, 2014 Comtrade data was analyzed against 2013 electricity reliability data and so forth. The two-year staggered model analyzed 2015 Comtrade data against 2013 electricity reliability data. Results for the one-year and two-year models matched the results of the direct year-to-year comparison. CAIDI NOR during the year-to-year comparison remained the strongest correlation. SAIFI MED was the mostly strongly correlated across all three scenarios. The appendix includes figures and tables associated with the one- and two-year staggered models.

### Qualitative analysis – electricity disruptions

Based on qualitative reports from Briggs and Stratton, Cummins, and Generac, however, consumers’ risk perception could explain the timing of generator purchases (Honda did not provide any reasons for variations in sales numbers). In one of its recent reports, Generac posited that increased outages and aging power grids contributed significantly to an increase in demand for backup power [18]. In February 2021, following the Texas winter freeze that resulted in prolonged power outages for millions of customers, Generac CEO Aaron Jagdfeld claimed that Generac could not manufacture generators quickly enough to meet demand [63]. In several of their reports, Briggs and Stratton asserted that generator sales depended on the number of power disruptions events, as stronger sales numbers correlated with more significant outages [50, 51]. In its 2017 report, for instance, the company claimed that Hurricane Matthew heightened demand for backup generators from regions affected by the hurricane [51]. As indicated above, a quantitative analysis of Comtrade data and reliability does not indicate a clear causal relationship between major event disruptions (MED) and demand. This may be due to the lack of regional specificity for the Comtrade data, which is not calibrated to specific regions in the U.S.

Other studies support generator companies reports by finding that major power outages could prompt an increase in private demand for backup generators. Conclusions from the Applied Technology Council found that public expectations of lifeline infrastructure systems, like electricity, seemed to deviate from these systems’ abilities to minimize disruptions [64]. The disparity between expectations and actual performance suggests that experiences of power outages could shift attitudes toward backup generators. Mayer et. al are more explicit in their assessment, which finds that 56% of business owners in South Texas attempted to purchase some form of backup generation following major power outages from Hurricane Rita in 2005. More than 2/3rd of respondents who attempted to purchase a backup generator did so [21].

### Quantitative analysis – macroeconomic indicators

**Fig. 7:**
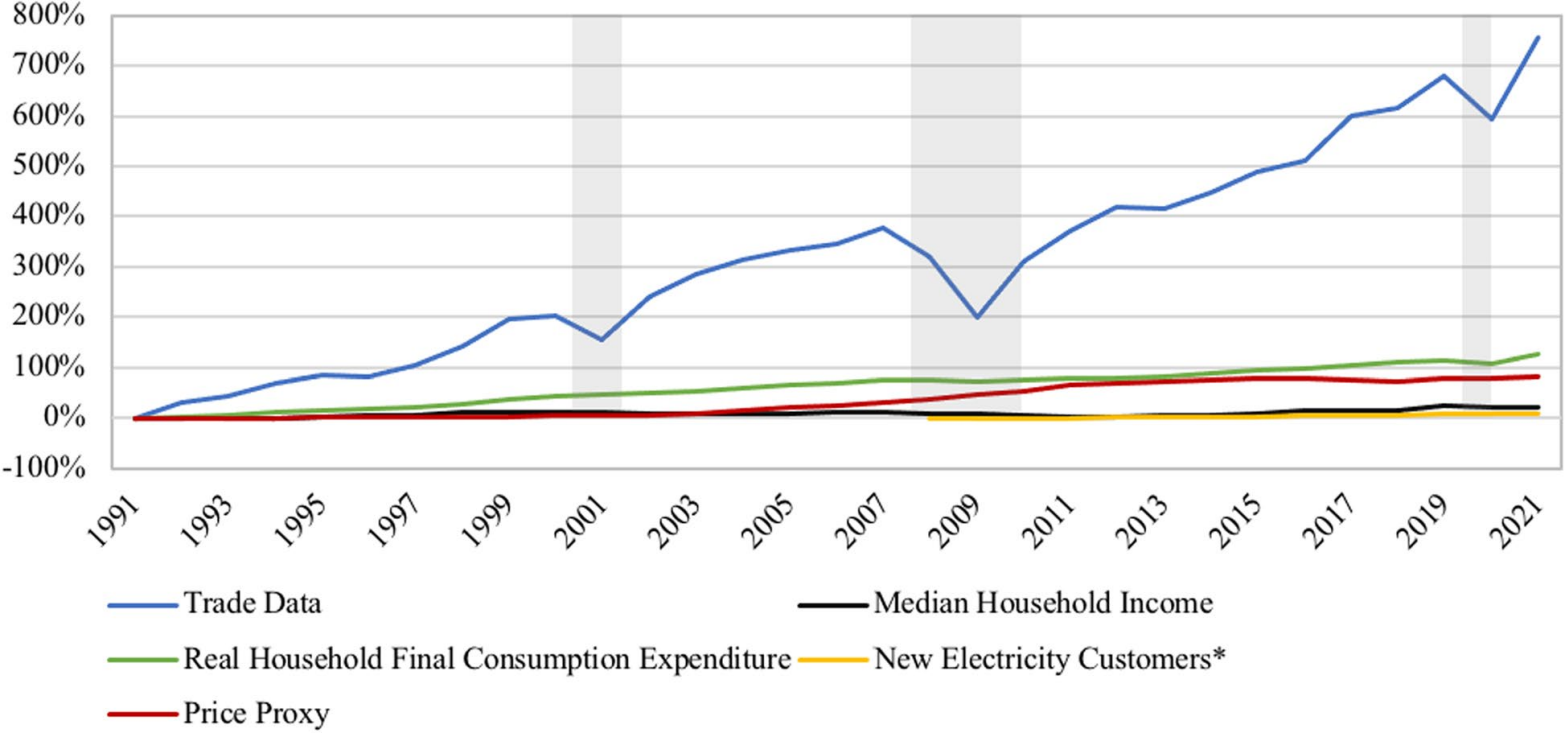
Change in Import Value of Backup Generators Compared to Change in Macroeconomic Indicators, 1991–2021. Shaded Areas Indicate U.S. Recession (FRED)

The historical trajectory of Comtrade data also was analyzed with macroeconomic indicators in a plot and regression analysis to determine if increases in trade data could be attributed to these indicators. Similar to the regression analysis of Comtrade data and reliability metrics, the coefficient and standard errors for all macroeconomic variables were large due to a disparity in the absolute numerical values between Comtrade data and the macroeconomic indicators. All macroeconomic indicators are strongly correlated with Comtrade data; however, none of these indicators explain the rate of growth in the import value of generators as indicated in Fig. 7, which suggests that consumers are demanding more generators than market growth would predict alone. These results also suggest that economic recession (consumers’ ability to pay) has an impact on sales data for backup generator demand (Table 5).

**Table 5:** Regression Analyses of Import Value of Backup Generators to Macroeconomic Indicators

Macroeconomic Indicator	Pearson Correlation	R^2^	Coefficient	Standard Error (Coefficient)	*p*-value
Median Household Income	0.744	0.553	-10,156,968,081	2,066,683,536	3.502E^−05^
Real Household Expenditure	0.969	0.938	-2,328,776,623	214,430,464	1.510E^−11^
Generator Price Proxy	0.890	0.792	-1,493,265,132	348,306,456	1.93698E^−4^
Number of Electricity Customers*	0.9432	0.890	-18,154,367,082	2,226,109,934	5.439E^−06^

* refer to the date range of 2008 - 2021 which is the only date range available for customer data

### Electricity disruption intolerance

Given that consumer demand for backup generators seems to have increased more quickly than other macroeconomic indicators, intolerance to power outages also could be driving demand. Esmalian et al. have defined this phenomenon for residential consumers as “the zone of tolerance,” or the measurable level of hardship on household well-being caused by power outages. In their theory, the zone of tolerance occupies the theoretical space between a household’s desired level of service (optimized normal) and an adequate level of service (a tolerable floor) [14]. Threshold analysis may help clarify why household demand in the U.S. may be changing in relation to power outages.

**Fig. 8:**
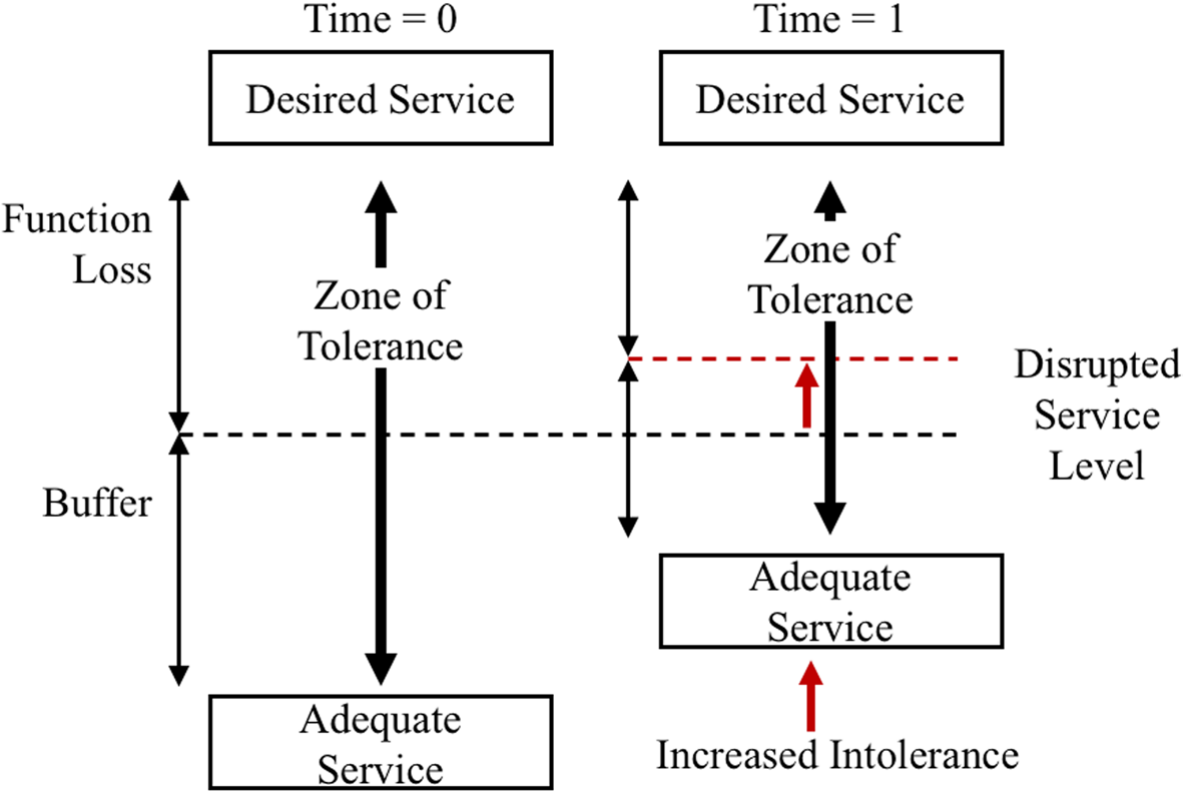
Increase in Intolerance to Electrical Outages, Using Model from Esmalian et. al 2021

Building from Esmailian’s conclusions and generator companies’ reports, this paper suggests that sales of backup generators may represent a manifestation of increasing household intolerance toward power outages. Perceived levels of need and a lack of service substitutability have increased household and business’s minimums for adequate levels of service, thereby increasing their levels of intolerance to a power outage, as depicted in Fig. 8. Over the last few decades there have been exponential increases in activities requiring electrical activities including banking, school, employment, healthcare, and entertainment. Generator purchases could indicate consumers’ ability to pay for energy redundancy—in terms of generator sales—in addition to their willingness to pay for energy redundancy measures.

Outage intolerance could help explain another major sales trend reported by generator companies. Results from the quantitative analysis of macroeconomic data do not support or contradict the theory of electrical intolerance as a driver in demand for backup generators. Rather, the data highlight that demand seems to have outstripped other macroeconomic data, suggesting the influence of another factor. The macroeconomic analysis serves to highlight an increase in historical demand over time but is insufficiently specific to apply to electrical intolerance. Reviewing qualitative statements from Cummins and Generac’s 2020 reports suggests that the concept of intolerance may help account for the rise in residential demand due to the Covid-19 pandemic. Although Cummins’s 2020 Annual Report observed that overall generator sales had declined, it noted an uptick in residential sales, as more employees worked from home that year. Generac similarly noted that residential sales had risen that year [18, 43]. As an increasing number of people migrated their essential functions almost entirely to at-home tasks that required electricity, residential intolerance to power outages rose, which may have contributed to increased demand for residential backup generators.

## Discussion

The results sections seem to find that consumers’ preferences for backup generators have increased in the U.S., which may be attributed partially to increased perceptions of risk and rising intolerance to electrical disruptions. Using historical trends as a template for future demand, results from this study suggest that a growing contingent of electricity consumers will play an increasingly active role in buying personal electrical resilience in the form of backup generators. This has implications for the second identified area of study on backup generators, which focuses on individual use of backup generators. The results suggest that studies in this field focused on the U.S. should consider the impact of an increase in private generator demand over on their conclusions. The implications of these results also may signal a shift in consumer behavior in countries like the United States, which presents a theoretical and practical opportunity to integrate this second area with of study with the first area of study on backup generators, which considers the importance of backup generators in supporting critical systems. Integrating these two areas of study more fully could increase individual and community electricity resilience in the U.S. by channeling consumer demand to support systemic resilience.

Although many U.S.-focused studies that examine individual demand for backup generators (second area of study) may not assume demand to be static, most do not account for a general increase in demand over time [19]. This could impact studies focusing on equity issues, as backup generators may exacerbate socioeconomic inequalities for residential consumers during a power outage [20]. Although more research is needed to determine which specific consumer segments purchase backup generators, high upfront costs of backup generators could make them prohibitive for lower-income customers. An increase in backup generator adoption by mostly middle to upper income consumers may widen a socioeconomic gap between people with and without power, which Esmalian et. al have noted [65].

Threshold and preparedness studies, in the second category of study, which examine the importance of backup generators for individuals could consider the potential impacts of long-run changes in individual demand on a collective or systemic level [13, 14]. Although backup generators have demonstrated utility as a buffer against power outages, the increased proliferation of certain types of backup generators, such as gasoline and diesel generators, could increase dependence on the inputs required for these types of generators. Consumers may be unable to purchase these materials due to the rising cost of these inputs, such as in the aftermath of the Texas winter storm Uri in 2021 [66]. Transportation failures during or following a disaster event also can limit consumers’ access to gasoline and diesel [67].

Studies of backup generators in commercial businesses seem to have paid more attention to these collective failure points. In the aftermath of superstorm Sandy, for instance, many businesses reported that they could not purchase gasoline due to shortages or price gouging, which crippled the efficacy of these generators [68]. The results of this study also seem to support the extant research on the use of backup generators in U.S. businesses, particularly Mayer et. al, by indicating that the results found in Mayer could present a case study for demands about risk perception in the U.S. more broadly [21].

The results of this paper also offer an opportunity to integrate private market demands into broader resilience systems more fully. Private demand for backup generators alone does not strengthen the resilience of electrical grids. As the results suggest, an increase in private demand suggests an increase in consumer dissatisfaction with the reliability of the electrical grid. Changes in private behaviors are not incorporated into studies on strengthening electricity networks, which tend to focus on critical facilities [12, 69]. Technical research on backup generators has begun to explore the possibilities of incorporating different types of backup generators into microgrid systems [9]. Other research and grey literature have taken an interest in the impact of generators on distributed systems [18, 20]. Determining the rate of backup generator demand, along with regional or other demographic variances in this demand, may allow researchers to build models to incentivize consumers to connect their generators to the electrical grid, which could increase systemwide resilience. Identifying the specifics of how to incorporate consumer demand electrical resilience is outside the scope of this paper, however, presenting an area of further research.

## Conclusion

This paper focuses on changes in consumer preference for backup generators in the U.S. to suggest that theories of energy resilience should incorporate changes in the private sector more fully. Determining the specifics of this demand may help electricity resilience studies develop mechanisms and incentives to encourage consumers to use backup generators as energy distribution sources. More specific sales data from backup generator companies could provide additional insight for these studies. Conducting targeted surveys may help shed additional light on causal factors shaping consumer demand [21, 70]. Studying utilities that have encouraged consumers to install and net meter backup generators also could serve as case studies to link private demand with collective action.

In addition, current U.S. trends in electricity resilience could be found in similar countries, which deserves further attention [71]. Strengthening the energy resilience in remote workspaces may be a part of this new horizon. As of this writing, several companies have invested in at-home backup generators for their employees [72]. These investments are too nascent to indicate a trend; however, they indicate a possible area for further study, especially if remote work remains an established norm for many businesses. This case also may provide an avenue to anticipate changes to other lifeline infrastructure systems, such as the privatization (and digitization) of aspects of the transportation network or of water.

## Data Availability

All datasets used in this study are publicly available. Source links for all data sets can be found below, along with in the references section. The methods section highlights how these data were used in the study. Backup Generator Company Reports: Briggs & Stratton (CIK 0,000,014,195). Backup Generator Company Reports: Cummins. Backup Generator Company Reports: Generac. Backup Generator Company Reports: Honda. UN Comtrade Database. Energy Information Administration (EIA) Power Reliability Data. EIA Number of Customers. St. Louis Federal Reserve Economic Data (FRED).
